# Association of eating habits with health perception and diseases among Chinese physicians: a cross-sectional study

**DOI:** 10.3389/fnut.2023.1226672

**Published:** 2023-08-11

**Authors:** Moxi Chen, Xuan Xu, Yinghua Liu, Ying Yao, Pianhong Zhang, Jingfang Liu, Qian Zhang, Rongrong Li, Hailong Li, Yan Liu, Wei Chen

**Affiliations:** ^1^Department of Clinical Nutrition, Peking Union Medical College Hospital, Chinese Academy of Medical Sciences & Peking Union Medical College, Beijing, China; ^2^The First Medical Center of PLA General Hospital of China, Beijing, China; ^3^Department of Clinical Nutrition, Tongji Hospital Affiliated to Tongji Medical College, Huazhong University of Science and Technology, Shanghai, China; ^4^The Second Affiliated Hospital Zhejiang University School of Medicine, Hangzhou, China; ^5^Division of Nutrition, National Clinical Research Center for Aging and Medicine, Huashan Hospital, Shanghai Medical College, Fudan University, Shanghai, China; ^6^Beijing Tongren Hospital, CMU, Beijing, China

**Keywords:** eating habits, health perception, disease, obesity, physicians

## Abstract

**Background:**

Some eating habits may be related to the development of gastrointestinal diseases, obesity, and related metabolic dysfunctions. Because of long working hours, and shift schedules, physicians are more likely to form such eating habits and have a high risk of developing these diseases.

**Objectives:**

We aimed to investigate the association between physicians’ eating habits and their health perception and diseases.

**Methods:**

Between 24 June and 5 August 2020, we performed convenience sampling of in-service physicians in hospitals in mainland China. A questionnaire was administered to collect data pertaining to basic sociodemographic characteristics, eating habits, health-related information such as body mass index classification, and prevalence of common diseases. The associations among eating habits and perceived suboptimal health status, micronutrient deficiency-related diseases, obesity, and related metabolic diseases were analysed.

**Results:**

The prevalence of unhealthy eating habits was high: more eating out-of-home (53.4% in hospital canteens, 23.0% in restaurants and takeaways), fewer meals at home, irregular meals (30.5%), and eating too fast (the duration <10 min, 34.6%). Among those with the above eating habits, the prevalence rates of sub-optimal health and disease were higher than among those without the above eating habits.

**Conclusion:**

Eating habits such as frequent eating out-of-home, irregular meals, and eating too fast were common among physicians, and were significantly related to perceived sub-optimal health status and disease occurrence.

## 1. Introduction

With the development of the social economy and improvement in the living standards, the eating habits of modern people have undergone tremendous changes compared to the past. These changes are not only reflected in the diversification of food sources and the complexity of processing them, but also in the consumption of fast-food and out-of-home meals. Excessive consumption of high-energy density and ultra-processed foods, fast food and takeaways, inadequate and irregular eating times, and snacking between meals are the main characteristics of the modern diet (1). Dietary patterns characterised by the consumption of red and processed meat, fast food, and sugar-sweetened beverages are considered to be important risk factors for chronic non-communicable diseases such as metabolic and cardiovascular diseases in Chinese residents (2), and the pathogenic effects of unhealthy eating habits are gradually attracting increasing attention.

The main manifestations of unhealthy eating habits are short and irregular meal times, and excessive consumption of fast food and takeaways. Eating too fast can affect the levels of gastrointestinal hormones such as ghrelin, YY peptide, and glucagon-like peptide-1 (GLP-1), and therefore disturb the balance between hunger and satiety (3). Thus, it can lead to redundant energy intake, and glucose and lipid metabolism disorders (4), which would in turn lead to an increased incidence of obesity, diabetes, and metabolic syndrome (MetS) (5). Eating too fast is also believed to be associated with gastrointestinal diseases such as indigestion (6). Out-of-home meals (canteens, fast food, takeaways, etc.) have been shown to be a risk factor for high energy and fat intake, and low micronutrient intake (especially vitamin C, calcium, and iron) (7). People with these habits show a higher prevalence of overweight and obesity (8).

Shift work is thought to be an important contributor to irregular meals in modern society. Shift workers exhibit a significant preference for nocturnal activity, higher intake of high-fat diets and sweets such as candy and sweetened beverages, and disordered temporal eating patterns (irregular timing and frequency of meals, night meals) (9). These unhealthy habits are thought to be associated with the elevation of cardiometabolic risk factors [higher triglyceride and lower high-density lipoprotein (HDL) levels] and increased incidence of chronic diseases (including MetS, obesity, diabetes, and hypertension) (10).

Medical personnel are one of the representative populations of shift work, and previous studies have reported that they have irregular diets and excessive intake of fast foods high in salt, fat, and/or sugar after night shifts (11). Because of the COVID-19 pandemic, physicians are confronted with more working pressure and irregular working arrangements (12). With the emerging problems of work-life balance, physicians are more likely to develop unhealthy eating habits such as fast eating and more out-of-home meals. Previous surveys on physicians’ the health statuses have found that the prevalence rates of obesity and related metabolic diseases [such as hyperlipidaemia and coronary artery disease (13)], and cardiovascular disease [such as hypertension (14)] were higher among physicians than in the general population. However, previous studies have mainly focused on the effects of physical inactivity (15) and sleep deprivation (16), and there are few studies on the association of physicians’ eating habits with their health perception and diseases.

Suboptimal health status (SHS) is a physical state between health and disease, characterised by perceived discomfort, general malaise, chronic fatigue, and low energy (17). Unhealthy eating habits, lack of physical activity, poor sleep quality, and other unhealthy living habits are significantly related to self-perceived SHS (18). We conducted this survey to explore the association between the physicians’ eating habits and their perceived SHS status, micronutrient deficiency-related diseases, obesity, and related metabolic diseases.

## 2. Subjects and methods

### 2.1. Subjects

From 24 June to 5 August 2020, we conducted a convenience sampling of in-service doctors in hospitals of all levels in mainland China (31 provinces, municipalities, and autonomous regions). Inclusion criteria were in-service physicians aged 20 years and above. Exclusion criteria were those with a physical or mental illness that affects eating habits (including eating behavior disorders, like bulimia and anorexia) and those who were unable to cooperate with the investigation. The online questionnaire was collected using mini apps in WeChat, and was forwarded to WeChat groups in different hospitals and departments to investigate the corresponding population. Each respondent voluntarily participated in the study and completed the questionnaire.

### 2.2. Questionnaire

The questionnaire we designed collected basic sociodemographic characteristics such as the age, sex and the department of physicians, eating habits, health-related information such as the body mass index (BMI) classification, perceived SHS status, and prevalence of common diseases. To rule out the influence of other lifestyle factors on the study results, we also included self-assessments of working time, sedentary time, exercise time and sleep quality. Smoking habits and alcohol consumption were also assessed through self-reported grading options, as detailed in the supplementary material (Original Questionnaire-Translated Version).

### 2.3. Eating habits

We divided eating habits into different types according to the source of meals, mode, and duration of eating. Specifically, the questionnaire counted the sources of the respondents’ working meals (hospital canteen, dining out, or home-made), frequency of meals at home in a week (< 7 times/week, 8–14 times/week, or ≥ 15 times/week), regularity of meals, and average meal duration (< 10 min/meal, 10–30 min/meal, > 30 min/meal).

### 2.4. Health perception and diseases

Participants reported the classification of their BMI (<18.5 kg/m^2^, 18.5 kg/m^2^ ≤ BMI < 24.0 kg/m^2^, 24 kg/m^2^ ≤ BMI < 28.0 kg/m^2^, and ≥ 28.0 kg/m^2^), which was graded according to the cut-off value for the Chinese population (19). And the assessment of their own health perception was recorded, which was graded to very healthy, basic health, subhealth, and disease state. In order to further evaluate the disease state of the participants, we also asked them to answer whether they often suffered from common diseases related to micronutrient deficiencies (cold, angular stomatitis/oral ulcer, chronic gastroenteritis/diarrhoea, constipation, and vision decrease/asthenopia), and whether they had obesity and metabolic-related chronic diseases (diabetes, cardiovascular and cerebrovascular diseases, dyslipidaemia, and tumours).

### 2.5. Quality control

The questionnaire used in this survey was designed on the basis of the “Report on Nutrition and Health Status of Chinese Physicians (2013)”, and its validity has been verified by a previous survey (20). The collected online questionnaires were reviewed in a timely manner. If key variables such as the name, sex, and work unit had logical errors or were missing, and could not be corrected and filled, the questionnaires were regarded as invalid.

### 2.6. Statistical analysis

Previous studies have shown that the prevalence of perceived poor or fair health status in physicians was approximately 21% (21); the type I error α was 0.05, and the permissible error δ was set at 0.02. Thus, at least 1,594 patients should be included in this study. All analyses were conducted with International Business Machines (IBM) Statistical Package for the Social Sciences (SPSS) version 26.0 (IBM Corp., Armonk, NY, United States). Categorical variables were described as absolute and relative frequencies, and comparisons between groups were performed using the chi-square test. Associations between eating habits and health perception [subhealth/disease compared with very/basically healthy (reference)] were analysed using multivariable logistic regression, while controlling for sociodemographic characteristics and other covariates (Model 1 was adjusted for sex and age; model 2 was adjusted for sex, age, working time, sleep quality, exercise time, sedentary time, smoking habits, and alcohol consumption). All statistical analyses were two-sided tests, and the null hypothesis was rejected when *p* < 0.05.

## 3. Results

### 3.1. General information

In total, 9,624 questionnaires were collected. After excluding 428 non-physicians and questionnaires with incomplete information, 9,196 questionnaires (95.6%) were recovered. The distribution of respondents among various regions was relatively even, comprising mainly of doctors from second-level and above hospitals, covering various clinical and administrative departments. Among all respondents, obstetricians and gynaecologists accounted for the highest proportion (3,710, 40.34%), followed by internal medicine (1,352, 14.7%), and nutrition specialists (1,027, 11.17%). Most were women (7,662, 83.32%), and were aged 30–50 years old (Table 1).

**Table 1:** Sociodemographic characteristics of the physicians.

Characteristics		*N*	%
Sex	Male	1,534	16.7
	Female	7,662	83.3
Age group (years)	20–29	1,350	14.7
	30–39	3,607	39.2
	40–49	2,809	30.5
	≥ 50	1,430	15.6
Hospital level	Tertiary	5,581	60.7
	Secondary	3,119	33.9
	Primary	496	5.4
Department	Gynaecology and obstetrics	3,710	40.3
	Internal medicine	1,352	14.7
	Nutrition	1,027	11.2
	Surgery	836	9.1
	Medical technology and administration	765	8.3
	Pediatrics	389	4.2
	Others	1,117	12.1
Main dining location	Hospital canteen	4,910	53.4
	Dining out	2,117	23.0
	At home	2,169	23.6
Frequency of consuming home-cooked meals	< 7 times/week	3,063	33.3
	8–14 times/week	4,019	43.7
	≥ 15 times/week	2,114	23.0
Eat regularly	Yes	6,395	69.5
	No	2,801	30.5
Eating duration	< 10 min/meal	3,186	34.6
	10–30 min/meal	5,794	63.0
	> 30 min/meal	216	2.3
Working time	< 40 h/week	1792	19.5
	40–60 h/week	5,628	61.2
	> 60 h/week	1776	19.3
Sleep quality	Good	2,492	27.1
	Average	5,358	58.3
	Poor	1,346	14.6
Exercise time	< 40 min/d	6,781	73.7
	40–60 min/d	1912	20.8
	> 60 min/d	503	5.5
Sedentary time	< 1 h/d	1,110	12.1
	1–2 h/d	1826	19.9
	> 2 h/d	6,260	68.1
Smoking habits	Yes	374	4.1
	No	8,822	95.9
Alcohol consumption	0 g/d	6,732	73.2
	1–14 g/d	2,197	23.9
	15–24 g/d	180	2.0
	≥ 25 g/d	87	0.9

### 3.2. Lifestyle

The prevalence of unhealthy eating and living habits among physicians was high, including eating out-of-home (53.4% in hospital canteens, 23.0% in dining out), less frequent meals at home, irregular meals (30.5%), and eating too fast (<10 min/meal, 34.6%). Those who worked more than 60 h per week accounted for 19.3% of the respondents. Poor sleep quality was reported by 14.6% of the physicians. In addition, physicians also had unhealthy lifestyle habits such as inadequate exercise (<40 min/d, 73.7%) and prolonged sedentary time (>2 h/d, 68.1%) (Table 1).

### 3.3. Health perception and diseases

**Figure 1:**
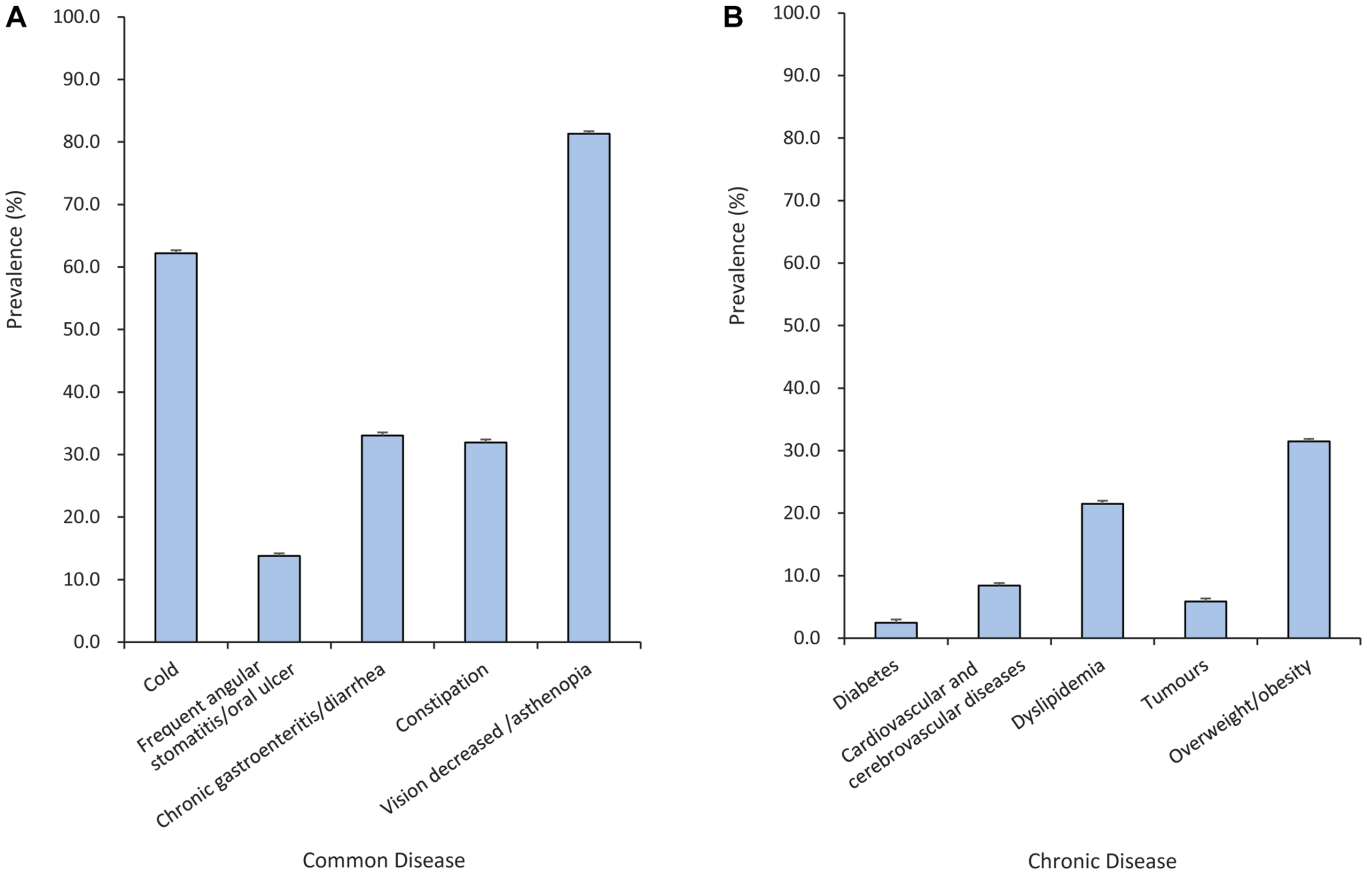
Prevalence of physicians’ self-reported common diseases **(A)** and chronic diseases **(B)**.

According to the BMI classification obtained from the survey, 31.5% of the physicians were overweight or obese (BMI ≥ 24 kg/m^2^), 56.6% thought they had SHS, 3.3% said they had diseases, and only 5.3% thought they were healthy. Among the physicians participating in the survey, the common diseases related to micronutrient deficiency ranked from high to low prevalence were decreased vision or asthenopia (81.3%), frequent colds (62.20%), chronic gastroenteritis or chronic diarrhoea (33.05%), constipation (31.94%), and frequent angular stomatitis/oral ulcer (13.80%); see Figure 1A. The prevalent obesity and metabolic-related chronic diseases were dyslipidaemia (21.50%), cardiovascular and cerebrovascular diseases (8.42%), tumours (5.87%), and diabetes (2.49%), as shown in Figure 1B.

### 3.4. Eating habits, health perception and diseases

Physicians with unhealthy eating habits, including when the working meals were mainly fast food and takeaways, few meals at home, irregular meals, and short meal duration (<10 min) had higher prevalence rates of micronutrient deficiency-related common diseases, obesity, and metabolic-related chronic diseases than other respondents (Table 2). After adjusting for the effects of sex, age, working hours, sleep quality, exercise time, sedentary time, smoking habits, and alcohol consumption, physicians with the above unhealthy eating habits was significantly associated with higher odds of subhealth/disease when compared with those with relatively healthy eating habits (Table 3). We further analyzed the differences in eating habits and their disease prevalence of physicians from different departments, and found that physicians from the department of nutrition had relatively healthy eating habits, that is, they mainly brought their own meals at work, had more meals at home, and had regular and sufficient eating time (Table 4). Correspondingly, nutrition doctors also have a relatively low prevalence of common diseases and chronic diseases than other departments (Table 5).

**Table 2:** Self-reported prevalence of common and chronic diseases among physicians by the different eating habits.

Eating habits	Common diseased *N* (%)					Chronic diseases N(%)				
	Cold	Frequent angular stomatitis/oral ulcer	Chronic gastroenteritis/diarrhoea	Constipation	Vision decrease/asthenopia	Overweight/obesity	Diabetes	Cardiovascular and cerebrovascular diseases	Dyslipidaemia	Tumours
Main dining location										
Hospital canteen	3,010 (61.3)***	674 (13.7)***	1,565 (31.9)***	1,522 (31.0)***	3,989 (81.2)***	1,460 (29.7)***	125 (2.5)***	418 (8.5)**	1,059 (21.6)***	274 (5.6)
Dining out	1,435 (67.8)	340 (16.1)	856 (40.4)	776 (36.7)	1788 (84.5)	834 (39.4)	44 (2.1)	165 (7.8)	491 (23.2)	130 (6.1)
At home	1,275 (58.8)	255 (11.8)	618 (28.5)	639 (29.5)	1701 (78.4)	602 (27.8)	60 (2.8)	191 (8.8)	427 (19.7)	136 (6.3)
Frequency of consuming home-cooked meals										
< 7 times/week	2004 (65.4)***	497 (16.2)***	1,158 (37.8)***	1,151 (37.6)***	2,583 (84.3)***	1,025 (33.5)**	69 (2.3)***	232 (7.6)***	670 (21.9)***	181 (5.9)
8–14 times/week	2,543 (63.3)	563 (14.0)	1,317 (32.8)	1,235 (30.7)	3,272 (81.4)	1,269 (31.6)	96 (2.4)	362 (9.0)	862 (21.4)	235 (5.8)
≥15 times/week	1,173 (55.5)	209 (9.9)	564 (26.7)	551 (26.1)	1,623 (76.8)	602 (28.5)	64 (3.0)	180 (8.5)	445 (21.1)	124 (5.9)
Eat regularly										
Yes	3,790 (59.3)***	726 (11.4)***	1771 (27.7)***	1780 (27.8)***	5,019 (78.5)***	1827 (28.6)***	155 (2.4)***	498 (7.8)***	1,307 (20.4)***	342 (5.3)**
No	1930 (68.9)	543 (19.4)	1,268 (45.3)	1,157 (41.3)	2,459 (87.8)	1,069 (38.2)	74 (2.6)	276 (9.9)	670 (23.9)	198 (7.1)
Eating duration										
< 10 min/meal	2076 (65.2)***	555 (17.4)***	1,271 (39.9)***	1,159 (36.4)***	2,740 (86.0)***	1,240 (38.9)***	96 (3.0)***	350 (11.0)***	827 (26.0)***	223 (7.0)**
10–30 min/meal	3,530 (60.9)	688 (11.9)	1716 (29.6)	1719 (29.7)	4,574 (78.9)	1,611 (27.8)	130 (2.2)	411 (7.1)	1,114 (19.2)	309 (5.3)
> 30 min/meal	114 (52.8)	26 (12.0)	52 (24.1)	59 (27.3)	164 (75.9)	45 (20.8)	3 (1.4)	13 (6.0)	36 (16.7)	8 (3.7)

Differences across the groups were tested with Pearson’s *χ*2 test: **p < 0.01, ***p < 0.001.

**Table 3:** Association between eating habits and health status.

Eating habits	Health status				Model 1			Model 2		
	Very/basically healthy		Subhealth/disease		OR	95% CI	*p*-value	OR	95% CI	*p*-value
Main dining location										
At home	1,011	46.6	1,158	53.4***	1.00			1.00		
Hospital canteen	2061	42.0	2,849	58.0	1.207	1.089–1.337	<0.001	1.117	1.001–1.247	<0.048
Dining out	618	29.2	1,499	70.8	2.057	1.807–2.342	<0.001	1.748	1.521–2.008	<0.001
Frequency of consuming home-cooked meals										
≥15 times/week	1,069	50.6	1,045	49.4***	1.00			1.00		
8–14 times/week	1,627	40.5	2,392	59.5	1.471	1.322–1.637	<0.001	1.237	1.103–1.388	<0.001
<7 times/week	994	32.5	2,069	67.5	2.038	1.813–2.292	<0.001	1.612	1.421–1.829	<0.001
Eat regularly										
Yes	3,072	48.0	3,323	52.0***	1.00			1.00		
No	618	22.1	2,183	77.9	3.234	2.918–3.584	<0.001	2.440	2.189–2.720	<0.001
Eating duration										
> 30 min/meal	131	60.6	85	39.4***	1.00			1.00		
10–30 min/meal	2,593	44.8	3,201	55.2	1.851	1.404–2.440	<0.001	1.420	1.058–1.905	0.019
<10 min/meal	966	30.3	2,220	69.7	3.549	2.677–4.705	<0.001	2.252	1.668–3.041	<0.001

Differences across groups were tested with Pearson’s *χ*^2^ test. ***p < 0.001. Associations were examined using multivariable logistic regression. Model 1 was adjusted for sex and age; model 2 was adjusted for sex, age, working time, sleep quality, exercise time, sedentary time, smoking habits, and alcohol consumption. subhealth/disease compared with very/basically healthy (reference). OR, odds ratio; CI, confidence interval.

**Table 4:** Eating habits among physicians from different departments.

Eating habits	Department *N* (%)							*p*-value
	Gynaecology and obstetrics	Internal medicine	Nutrition	Surgery	Medical technology and administration	Pediatrics	Others	
Main dining location								<0.001
Hospital canteen	1975 (53.2)	672 (49.7)	632 (61.5)	457 (54.7)	438 (57.3)	196 (50.4)	540 (48.3)	
Dining out	846 (22.8)	407 (30.1)	107 (10.4)	261 (31.2)	135 (17.6)	98 (25.2)	263 (23.5)	
At home	889 (24.0)	273 (20.2)	288 (28.0)	118 (14.1)	192 (25.1)	95 (24.4)	314 (28.1)	
Frequency of consuming home-cooked meals								<0.001
< 7 times/week	1,332 (35.9)	492 (36.4)	222 (21.6)	351 (42.0)	213 (27.8)	120 (30.8)	333 (29.8)	
8–14 times/week	1,662 (44.8)	585 (43.3)	411 (40.0)	352 (42.1)	321 (42.0)	176 (45.2)	512 (45.8)	
≥15 times/week	716 (19.3)	275 (20.3)	394 (38.4)	133 (15.9)	231 (30.2)	93 (23.9)	272 (24.4)	
Eat regularly								<0.001
Yes	2,286 (61.6)	922 (68.2)	984 (95.8)	478 (57.2)	653 (85.4)	252 (64.8)	820 (73.4)	
No	1,424 (38.4)	430 (31.8)	43 (4.2)	358 (42.8)	112 (14.6)	137 (35.2)	297 (26.6)	
Eating duration								<0.001
< 10 min/meal	1,573 (42.4)	449 (33.2)	157 (15.3)	353 (42.2)	206 (26.9)	103 (26.5)	341 (30.5)	
10–30 min/meal	2074 (55.9)	870 (64.3)	826 (80.4)	465 (55.6)	535 (69.9)	278 (71.5)	746 (66.8)	
> 30 min/meal	63 (1.7)	33 (2.4)	44 (4.3)	18 (2.2)	24 (3.1)	8 (2.1)	30 (2.7)	

Differences across the groups were tested with Pearson’s *χ*^2^ test.

**Table 5:** Self-reported prevalence of common and chronic diseases among physicians from different departments.

Diseases	Department *N* (%)							*p*-value
	Gynaecology and obstetrics	Internal medicine	Nutrition	Surgery	Medical technology and administration	Pediatrics	Others	
Common diseases								
Cold	2,423 (65.3)	861 (63.7)	552 (53.7)	501 (59.9)	460 (60.1)	242 (62.2)	681 (61.0)	<0.001
Frequent angular stomatitis/oral ulcer	604 (16.3)	178 (13.2)	77 (7.5)	116 (13.9)	99 (12.9)	61 (15.7)	134 (12.0)	<0.001
Chronic gastroenteritis/diarrhoea	1,333 (35.9)	448 (33.1)	218 (21.2)	292 (34.9)	240 (31.4)	122 (31.4)	386 (34.6)	<0.001
Constipation	1,425 (38.4)	407 (30.1)	200 (19.5)	210 (25.1)	209 (27.3)	125 (32.1)	361 (32.3)	<0.001
Vision decrease/asthenopia	3,189 (86.0)	1,104 (81.7)	745 (72.5)	649 (77.6)	610 (79.7)	315 (81.0)	866 (77.5)	<0.001
Chronic diseases								
Overweight/obesity	1,192 (32.1)	423 (31.3)	185 (18.0)	329 (39.4)	257 (33.6)	128 (32.9)	382 (34.2)	<0.001
Diabetes	92 (2.5)	28 (2.1)	27 (2.6)	29 (3.5)	20 (2.6)	5 (1.3)	28 (2.5)	<0.001
Cardiovascular and cerebrovascular diseases	330 (8.9)	96 (7.1)	61 (5.9)	107 (12.8)	60 (7.8)	27 (6.9)	93 (8.3)	<0.001
Dyslipidaemia	762 (20.5)	299 (22.1)	196 (19.1)	234 (28.0)	159 (20.8)	79 (20.3)	248 (22.2)	<0.001
Tumours	296 (8.0)	58 (4.3)	35 (3.4)	41 (4.9)	36 (4.7)	17 (4.4)	57 (5.1)	<0.001

Differences across the groups were tested with Pearson’s *χ*^2^ test.

## 4. Discussion

Our survey results showed that unhealthy eating habits, including more out-of-home eating, fewer meals at home, irregular meals, and eating too fast were common among physicians. These eating habits were significantly related to the perceived sub-optimal health status and disease occurrence, and even after adjusting for the effects of confounding factors such as sex, age, sleep quality, and exercise on the health perception, the risk of developing SHS and disease among physicians with the above eating habits remained significantly elevated. Nutrition doctors, because of work or life factors, have relatively healthy eating habits and a relatively low prevalence of common and chronic diseases, indicating the importance of healthy eating habits and eating environment for physicians to maintain health.

Previous studies generally believe that physicians have better health awareness and health habits than ordinary people, so they mainly focus on their mental stress and burnout. In recent years, studies have found that the incidence of diseases related to overweight, obesity (16) and dyslipidemia (13) in physicians may be significantly higher than that in the general population. This is significantly related to their long working hours, lack of physical activity and the shift system (21). A study investigating the health habits of police officers, ambulance workers, hospital staffs (doctors and nurses) and office workers found that doctors had significantly higher dining-out rates than those in other jobs, and skipped breakfast more often than office workers (22).

Dining out and fast-food rich diets are often characterised by high-energy density, high-fat, larger food portions, and higher consumption of sugar-sweetened beverages (23), while home-cooked meals involve healthier food preparation methods and more dietary varieties (24). Studies in adolescents and adults have demonstrated that increasing the number of meals at home can improve diet quality and reduce obesity and related diseases (25) due to increased fruit/vegetable and dairy consumption and reduced consumption of sugar-sweetened beverages and unhealthy snacks (26). Our study found that physicians had more meals outside and fewer meals at home, consistent with the results of previous studies (27–29), which demonstrated physicians’ high consumption of fast food and alcoholic beverages and less intake of fruits and vegetables.

Due to irregular meals and limited supply time in hospital canteens (30), physicians usually eat faster and tend to consume more snacks at unusual times (mainly at night) (31). These unhealthy eating habits collectively destroy their energy balance by increasing the serum concentrations of appetite-stimulating hormones (e.g., insulin, glucagon-like peptide 1, and YY peptide) and reducing diet-induced thermogenic effects (32), thus raising the risk of obesity and related metabolic diseases. Circadian rhythm disturbances caused by shift work and excessive intake of ultra-processed foods have also been linked to decreased immune function (33), chronic gastrointestinal dysfunction (34), and increased incidence of breast cancer (35), colorectal cancer (36), and other diseases. These findings are consistent with our results that showed that irregular meals and a short meal duration are associated with an increased prevalence of colds, chronic gastroenteritis/diarrhoea, and tumours.

Our study also found that there were significant differences in eating habits and health status among physicians from different departments. Doctors in nutrition departments had relatively healthy eating habits, and the corresponding prevalence rates of common and chronic diseases were low. This may be related to their relatively regular working hours, day shift work, and relatively comprehensive health knowledge (37). In previous studies, researchers investigated the differences of health habits among people of different occupational types in hospitals, and found that nurses had relatively worse health habits and health status than doctors which was also related to their long working hours, shift work and irregular work schedule (38). Other studies have also shown that the supplying timing of hospital canteens and the quality of meals (30) are important factors hindering physicians’ pursuit of healthy diets.

To the best of our knowledge, the present study is the first to investigate the relationship between the eating habits of physicians and their health perception and diseases. The results suggest a relationship between the increased prevalence of SHS and disease among physicians and their work patterns and eating habits, which may be related to the hospitals’ eating environment. This relationship indicates the necessity of paying attention to physicians’ working pressure and improving the supply of healthy food in hospital canteens to improve the health status and work efficiency of physicians. The “Healthy Canteens” initiative promoted by the Chinese National Health Commission is a meaningful attempt in this direction.

This study also has some limitations. Although the survey covered many departments, obstetrics and gynaecology, internal medicine, and nutrition accounted for more than 60% of the participants, which led to a sex imbalance (women accounted for 83.32% of respondents). Thus, this sample might underrepresent the male-dominated population of surgeons. In addition, Self-reported BMI is somewhat inaccurate, but among physicians it can be calculated more professional and frequent, because doctors usually pay more attention to their health status. Our study was in the form of an online questionnaire, so the participants’ eating habits, health perception and diseases were all self-reported. Thus, information bias cannot be ignored, even if the questionnaires were self-assessments conducted by trained physicians themselves. However, the validity of our questionnaire has been verified by previous survey (20) and can minimize the deficiency of self-reported information.

## 5. Conclusion

Eating habits such as frequent eating out-of-home, irregular meals, and eating too fast were common among physicians. These eating habits were significantly related to the occurrence of perceived SHS, micronutrient deficiency-related diseases, obesity, and related metabolic diseases diseases.

## Data availability statement

The original contributions presented in the study are included in the article/Supplementary material, further inquiries can be directed to the corresponding authors.

## Ethics statement

Ethical approval was not required for the studies involving humans because this is an observational survey study. Written informed consent for participation was not required from the participants or the participants’ legal guardians/next of kin in accordance with the national legislation and institutional requirements because the questionnaire was filled in anonymously online.

## Author contributions

WC contributed to conception and design. YiL, YY, PZ, JL, and QZ contributed to data collection. RL and HL supervised the study. YaL and XX contributed to statistical analysis and interpretation of data. MC contributed to data interpretation and manuscript drafting. All authors contributed to the article and approved the submitted version.

## Funding

This work was supported by the grants from National Key R & D Program of China (grant number: 2020YFC2005000).

## Conflict of interest

The authors declare that the research was conducted in the absence of any commercial or financial relationships that could be construed as a potential conflict of interest.

## Publisher’s note

All claims expressed in this article are solely those of the authors and do not necessarily represent those of their affiliated organizations, or those of the publisher, the editors and the reviewers. Any product that may be evaluated in this article, or claim that may be made by its manufacturer, is not guaranteed or endorsed by the publisher.
