# Supplementary material for: Association of eating habits with health perception and diseases among Chinese physicians: a cross-sectional study
Source: Front Nutr. 2023 Aug 11;10:1226672. doi: 10.3389/fnut.2023.1226672 (PMC10452877; doi:10.3389/fnut.2023.1226672)
Supplement: Supplementary file 1 [file Data_Sheet_1.docx]

**Translated version**

**PartⅠ general information**

1. Name (initials)

2. Province

3. The level of hospital

1. Non-physicians
2. Tertiary-level hospitals
3. Second-level hospitals
4. Community health service institutions
5. Township health institutions

4．Gender

1. Male
2. Female

5. Department

1. Internal medicine
2. Surgery
3. Obstetrics and gynecology
4. Department of nutrition
5. Medical technology department
6. Community general practice
7. Oncology department
8. Administrative and functional departments
9. Pediatrics
10. Emergency department
11. Health care department
12. Geriatric department
13. Other departments

6. Working age

1. Non-medical worker
2. Less than 5 years
3. 5-10 years
4. 10-15 years
5. More than 15 years

**PartⅡ Physical conditions, work and life routine**

1. Body Mass Index（BMI）

1. 28.0 or higher
2. 24.0 ≤ BMI < 28.0
3. 18.5 ≤ BMI < 24.0
4. < 18.5

2. What is your main source of working meals?

1. Hospital canteen
2. Home-cooked meals
3. Dining out, fast food mainly

3. How many meals at home per week?

1. Less than 7 times
2. 8 to 14 times
3. 15 times or more

4. Whether your meals regularly？

1. Often can't eat on time.
2. Basically regular.

5. How long do you eat your working meals?

1. Within 10 minutes
2. 10-30 minutes
3. More than 30 minutes

6. How many hours do you work per week?

1. Within 40 hours
2. 40-60 hours
3. More than 60 hours

7. How much sleep do you get?

1. Less than 4 hours/day
2. 4-7 hours/day
3. >7 hours/day

8. Your sleep quality

1. good
2. Average
3. Not good

9. How much exercise do you do?

1. Less than 40 minutes a day
2. 40 minutes to an hour a day
3. More than 1 hour a day

10. How much sedentary time do you spend on ongoing work?

1. Within 1 hour
2. 1-2 hours
3. More than 2 hours

11. Smoking status

1. Non-smoking
2. Have the habit of smoking

12. Alcohol consumption

1. Never drink alcohol
2. Less than 15g of alcohol per day (equivalent to 450mL of beer)
3. Less than 25g of alcohol per day (equivalent to 750mL of beer)
4. Daily alcohol intake ≥25g

13. How much time do you get sun exposure each day?

(Sun exposure means exposure to the sun outside without sunscreen)

1. No or no fixed time
2. Less than 30 minutes
3. ≥30 minutes

**Part Ⅲ Nutrients intake**

According to the recommendation of‘Dietary Guidelines for Chinese Residents (2016)’ for the 1800-2000Kcal energy intake, the types and quantities of required foods are shown in the title. Based on this estimate, your daily intake of different food groups is approximately (select the number of grams closest to your daily intake)

1. The weight of cereals, such as rice and noodles, per day (recommended amount is 250g)

1. 0g
2. ~50g
3. ~100g
4. ~150g
5. ~200g
6. ~250g
7. ~300g
8. ~350g
9. ~400g
10. ~450g
11. ~500g or above

2. The weight of vegetables, such as rape, celery, etc. per day (recommended amount is 450g)

1. 0g
2. ~50g
3. ~100g
4. ~150g
5. ~200g
6. ~250g
7. ~300g
8. ~350g
9. ~400g
10. ~450g
11. ~500g
12. ~550g
13. ~600g
14. ~650g
15. ~700g
16. ~750g or above

3. The weight of fruit, such as apples, oranges, etc., per day (recommended 300g)

1. 0g
2. ~50g
3. ~100g
4. ~150g
5. ~200g
6. ~250g
7. ~300g
8. ~350g
9. ~400g
10. ~450g
11. ~500g or above

4. The weight of soy, such as soybeans, per day (recommended amount is 15g)

1. 0g
2. ~20g
3. ~30g
4. ~40g
5. ~50g
6. ~60g
7. ~70g
8. ~80g
9. ~90g
10. ~100g
11. ~111g or higher

5. Daily intake of nuts, such as sunflower seeds, peanuts, etc. (recommended amount is 10g)

1. 0g
2. ~5g
3. ~10g
4. ~15g
5. ~20g
6. ~25g
7. ~30g
8. ~35g
9. ~40g
10. ~45g
11. ~50g or above

6. Daily intake of dairy products, such as milk (recommended amount is 300g, about 300~375mL)

1. 0mL
2. ~50mL
3. ~100mL
4. ~150mL
5. ~200mL
6. ~250mL
7. ~300mL
8. ~350mL or above

7. The weight of animal and poultry meat, such as fillet, chicken leg, etc., per day (recommended amount is 50g)

1. 0g
2. ~50g
3. ~100g
4. ~150g
5. ~200g
6. ~250g
7. ~300g
8. ~350g
9. ~400g
10. ~450g
11. ~500g or above

8. The weight of eggs, such as eggs, per day (recommended amount is 50g)

1. 0
2. ~1 PCS
3. ~2 PCS
4. ~3 PCS
5. ~4 PCS
6. ~5 or more

9. Daily intake of aquatic products, such as grass carp, shrimp (recommended amount is 50g)

1. 0g
2. ~50g
3. ~100g
4. ~150g
5. ~200g
6. ~250g
7. ~300g
8. ~350g
9. ~400g
10. ~450g
11. ~500g or above

**Part Ⅳ Health conditions**

1. Do you know your own bone density?

1. Unclear
2. Basically normal
3. Low bone mass
4. Osteoporosis

2. How many times have you had a common cold in a year?

1. Hardly a cold
2. 3 times or less
3. More than 3 times

3. Do you often suffer from keratitis or oral ulcer?

1. Often
2. Occasionally
3. Never

4. Do you have chronic gastroenteritis or chronic diarrhea?

1. Yes
2. No

5. Are you often constipated?

1. Yes
2. No

6. Do you have diabetes?

1. Yes
2. No
3. Unclear

7. Do you have cardiovascular and cerebrovascular diseases (such as hypertension, coronary heart disease, etc.)?

1. Yes
2. No
3. Unclear

8. Are your blood lipids normal?

1. Yes
2. No
3. Unclear

9. Do you have visual impairment or visual fatigue?

1. Yes
2. No

10. Do you have a known tumor?

1. Yes
2. No

11. What do you think of your current health?

1. Very healthy
2. Basic health
3. Subhealth
4. Disease state
